# Exploring the Need and Benefits of Digital Therapeutics (DTx) for the Management of Heart Failure in India

**DOI:** 10.7759/cureus.49628

**Published:** 2023-11-29

**Authors:** Balbir Singh, Prakash Hazra, Sanjeeb Roy, Rajeev Garg, Sanjay Bhat, Nitin Patki, Chetan Gharat, Kamlesh Patel, Jeeten Tandel

**Affiliations:** 1 Cardiology, Max Super Speciality Hospital, Saket, Delhi, IND; 2 Cardiology, Advanced Medicare and Research Institute (AMRI) Hospitals, Kolkata, IND; 3 Cardiology, ManglamPlus Medicity, Jaipur, IND; 4 Cardiology, Aware Gleneagles Global Hospitals, Hyderabad, IND; 5 Cardiology, Aster CMI Hospital, Bengaluru, IND; 6 Cardiology, Jupiter Hospital, Pune, IND; 7 Medical Affairs, Lupin Digital Health Limited, Mumbai, IND

**Keywords:** remote monitoring, readmissions, medication adherence, patient education, cardiologist

## Abstract

Indian heart failure (HF) registries consistently indicate high hospital readmissions and increased mortality rates after HF diagnosis. The challenges of Indian cardiologists in HF management include limited longitudinal data, frequent readmissions, low medication adherence, inadequate monitoring and follow-up, insufficient patient education, and lack of standard guidelines on cardiac rehabilitation. This article outlines the adoption of digital therapeutics (DTx) in HF management as a potential solution to address these challenges. DTx services offer improved medication adherence, early symptom identification, remote vital monitoring, timely intervention, patient education on symptoms, self-awareness, and lifestyle. Overall, DTx for HF comprises a dedicated team of cardiologists, health coaches, care managers, and globally certified connected devices to provide comprehensive and proactive monitoring, personalized coaching and support, behavioral engagement to improve adherence, emergency response system, delivery of medications and diagnostic tests at home, and a dedicated application for caregivers. DTx has the potential to enhance HF management in India.

## Introduction and background

Heart failure (HF) is a complicated medical condition characterized by the inability of the heart to adequately pump the necessary amount of blood to fulfill the body's needs, with an underlying issue that hampers the ability of the heart to eject blood in systemic circulation [1]. It affects a staggering 64.5 million people worldwide, with an annual diagnosis rate of one million patients, with India accounting for 1% of the total population (10-12 million) [2]. Indians have a higher risk of hospitalization (2-4 times more) for complications related to coronary artery disease (CAD) compared to other ethnic groups. Additionally, admission rates for Indians under 40 are significantly higher, ranging from five to 10 times more [3]. Evidence from Indian HF registries is limited but consistently projects the high burden of hospital readmissions and longitudinal increase in the mortality rates post-initial diagnosis in individuals affected with HF [4].

The primary objectives of management of this chronic heart disease involve alleviating symptoms, preventing hospitalization, and enhancing prognosis. Clinical trials have primarily focused on these three aspects of HF management [5]. Despite various advancements in the management of HF, cardiologists still face various challenges. These include limited vital and diagnostic data, low adherence rates, frequent readmissions, absence of standardized cardiac rehabilitation, diet and exercise plans, individualized lifestyle guidance, insufficient patient awareness and education, lack of understanding regarding the impact of poor adherence on heart health, delayed counseling seeking, and high risk of hospitalizations [6-8]. Addressing these challenges is crucial for improving prognostic outcomes.

The COVID-19 pandemic has accelerated the adoption of digital health solutions for monitoring HF patients in outpatient settings [9]. Implementing remote patient management for this group of patients can assist in identifying early indications and symptoms of cardiac decompensation. This, in turn, facilitates the prompt initiation of suitable treatment and care before the onset of HF decompensation. However, implementing digital advances to address the abovementioned challenges might be difficult. Additionally, when it comes to HF, a key challenge in developing digital health solutions lies in the presence of additional comorbidities such as diabetes, hypertension, renal failure, or chronic obstructive pulmonary disease. Each of these comorbidities has its guidelines for patient management, which introduces added complexity when designing digital therapeutics (DTx) algorithms. Aligning these diverse recommendations can be challenging as they may not always perfectly align with one another, posing a hurdle in the development of DTx solutions for HF.

Digital health incorporates diverse technologies that engage patients in managing their health, including mHealth, telehealth, smart devices, sensors, wearables, health information technology, and personalized medicine. Application-based DTx aims to improve overall health or target HF. Prior research demonstrates the potential for enhancing the quality of life (QOL), contingent upon the intervention design and application efficacy [10]. DTx services offer numerous advantages, including improved medication adherence, early symptom identification, remote vital monitoring, timely diagnostics, intervention by connecting patients with treating cardiologists for abnormal vitals and laboratory results, scheduled follow-ups, patient education on symptoms, triggers, self-awareness, diet, lifestyle, and exercise. These services also have the potential to lower drug manufacturing costs and provide insurers with benefits through personalized product customization. However, DTx adoption in India remains limited, with only a few companies considering investments and entry into this field [11].

This article outlines the unmet need for optimal management of HF in India and further emphasizes the benefits of DTx in heart care.

## Review

Current challenges faced by cardiologists in HF management in India

Cardiologists practicing across India face numerous challenges when managing patients with HF. The scarcity of data, especially longitudinal data regarding HF management in India, is a major challenge for Indian cardiologists. This issue hampers their understanding of the possible reasons for higher rates of readmissions and mortality. At present, there is only one study from India that reported longitudinal five-year follow-up data in patients with HF. High rates of hospital readmissions in patients with HF are another key concern raised by Indian cardiologists. The readmission rates at one year and five years were 30.2% and 49% in Trivandrum Heart Failure Registry, respectively [4,12]. The observations from the National Heart Failure Registry reported 8.4% readmission rates in patients with acute decompensated HF [6]. High readmission and mortality rates in the first year of post-hospitalization follow-up are reported in different HF registries from India [6,12]. Several factors have been identified as contributing to increased rates of hospital readmissions and mortality in India among individuals with HF. These factors include insufficient utilization of guideline-directed medical therapy (GDMT), inadequate education and awareness, non-adherence to treatment guidelines, poor compliance with prescribed treatments, advanced age, and being classified as New York Heart Association (NYHA) functional class IV.

Hospital readmissions in patients with HF increase the risk of mortality, and a significant proportion of these readmissions can be avoided. Taking steps to prevent hospital readmissions has the potential to greatly enhance the prognostic outcomes for HF patients in India. Implementing care coordination, team-based care, telemonitoring, and interventions focusing on medication adherence can effectively decrease the risk of hospital readmissions and mortality associated with HF.

A strong wall of evidence across the world indicates that there is low awareness and knowledge about HF among patients with HF. Few patients are unsure of the presence of HF, indicating a lack of awareness about their condition, and few lack knowledge about how to self-assess HF symptoms, which is an important skill for managing the condition and seeking appropriate medical care. Another concerning issue is that some of the patients are unsure of the purpose of HF medications. This lack of understanding could lead to poor adherence to medication regimens and suboptimal management of HF [13-15]. Overall, a significant proportion of patients with HF do not receive adequate education about their condition, possibly explaining the lack of knowledge. These factors imply a need for improved HF education programs to ensure that patients are well-informed about their condition, its symptoms, and the purpose of their medications. Providing patient education and proper counseling sessions for patients and their families to manage HF at home can decrease the frequency of unscheduled visits and readmissions, which are significant contributors to overall healthcare expenses [15].

Patient adherence, or adherence to treatment plans, is a critical factor in managing various chronic conditions, including HF. Heart failure is a complex condition that requires long-term management and adherence to treatment recommendations to optimize patient outcomes and reduce the risk of complications. Insufficient adherence to prescribed therapeutic regimens poses a significant barrier to achieving favorable clinical outcomes within the HF population. Current adherence rates for recommended medications, low-sodium diets, and aerobic exercise programs fall below the threshold necessary to reduce the morbidity and mortality associated with HF [7]. A review of published qualitative research studies indicated that a perceived lack of support, concerns about taking medication, and lack of engagement in exercise and lifestyle changes were the key barriers to achieving better adherence to treatment plans among people with cardiovascular diseases [16]. A recent literature review reported the five groups of cardiometabolic patient-related factors that influence patients' adherence to overall treatment: (1) health beliefs, knowledge, and perceptions regarding the risks and challenges of disease and medication intake along with adherence process perceptions, (2) self-concept, (3) emotions, (4) patient-healthcare provider relationship/communication, and (5) social and cultural interactions [17].

Non-adherence to medication is a critical problem, particularly prevalent in developing countries such as India, where patients often attempt to cut costs by discontinuing their medications without seeking medical advice. However, it is crucial to recognize that failing to comply with prescribed drugs can have severe consequences, including life-threatening situations such as acute pulmonary edema, while also resulting in increased financial burdens [15]. Ultimately, patient adherence in HF is a collaborative effort between healthcare providers, patients, and their support systems. By prioritizing education, open communication, and patient empowerment, healthcare teams can improve adherence rates, leading to better outcomes and quality of life for individuals with HF.

Heart failure is a serious medical condition that requires diligent monitoring and regular follow-ups. Without appropriate management and care, the consequences of HF can be severe and potentially catastrophic. Indian people affected with HF fail to continue close monitoring of their health condition regularly and miss follow-up visits. The major reasons could be cost expenditure associated with post-discharge follow-up and lack of knowledge and education about the severe consequences of neglecting regular monitoring and follow-ups. Observation from the Manipal Heart Failure Registry suggested that engaging in fluid and salt restriction while monitoring weight was associated with reduced mortality rates and lower rates of recurrent hospital admissions [15]. It is vital to monitor weight, blood pressure (BP), pulse, salt and fluid intake, and dose titrations of drugs to prevent complications.

Additionally, there are no Indian consensus guidelines that mention specific cardiac rehabilitation practices, diet, and exercise plans to implement, particularly in patients with HF. Hence, there is uncertainty among cardiologists about the use of cardiac rehabilitation programs, exercise plans, and diet.

Benefits of digital therapeutics

Digital therapeutics exhibits the potential to enhance primary care practice by enabling physicians to deliver treatment beyond the confines of a clinic or hospital, thereby addressing the financial burden and cost-related challenges in healthcare, particularly notable in regions such as India. This advantage allows for flexibility in providing treatment anywhere and at any time, offering a solution to poor adherence due to financial constraints. Primary care physicians can leverage technological innovations to offer cost-effective treatment options that address existing treatment gaps. This, in turn, promotes collaboration between patients and healthcare providers to attain improved health outcomes [18]. Initial findings from the study on DTx indicate favorable effects on overall well-being, rates of hospitalization, and enhancement of medication utilization among patients [9]. Digital therapeutics can offer several benefits in the management of chronic diseases [19-23]. This modern technology can assist patients by providing them with resources that promote modifications in their lifestyle, enhance their understanding of medical conditions, encourage self-care practices, and guide personalized treatment decisions. As a result, utilization of this technology has the potential to enhance overall well-being, prevent unnecessary expenses, and improve health outcomes [10]. In patients with HF, DTx can enable continuous remote monitoring of patients, allowing healthcare providers to track vital signs, symptoms, and medication adherence outside of traditional clinical settings. This real-time data helps detect early warning signs, prevent exacerbations, and optimize treatment plans. DTx can empower patients to take an active role in managing their condition by providing them with tools, resources, and personalized insights. This engagement promotes a sense of ownership, improves self-care behaviors, and leads to better health outcomes. As the integration of DTx progresses, it holds the potential to modernize the healthcare industry by offering accessible and cost-effective solutions.

DTx for HF overview

DTx for HF must have an evidence-based holistic heart care program that significantly reduces the risk of a heart attack and improves vitals and quality of life for cardiac patients. This DTx solution can help patients improve their heart health through doctor-connected online and offline modules. DTx for HF can enable seamless monitoring of patient vitals and can intervene to assist and guide patients during emergencies. Coupled with a host of features and designed with inputs from leading cardiologists, DTx for HF can improve the QOL of patients and reduce the stress and anxiety of their caregivers. DTx for HF will aid in solving the aforementioned issues in HF management in India.

Modules for Comprehensive Care

DTx for HF consists of six modules of comprehensive HF care.

Comprehensive and proactive monitoring: The first module deals with proactive monitoring through smart devices, a six-minute walk test, and symptom reporting. Doctors will have access to weekly digital reports and have an early warning system to predict acute decompensation. Key vitals and symptoms will be checked periodically via connected devices such as a smartwatch with BP and heart rate (HR) monitor, weighing scale, glucose monitor, etc. The six-minute walk test will be conducted at prescribed intervals with results visible to the doctor, and a comprehensive suite of laboratory tests with home pickup will also be conducted.

Personalized coaching and support: In this module, personalized coaching for lifestyle changes will be suggested. Sessions with health coaches will be planned to help the patient manage his/her disease through a personalized diet and exercise plan, designed based on the patient's lifestyle, condition, and preference. Furthermore, this module will also have health education sessions on the disease through videos and health articles/blogs to continuously educate the patient and caregivers. Lastly, dedicated care managers will be present through the DTx to ensure adherence and assist with this new technology.

Driving behavioral change for adherence: The third module will focus on behavioral engagement to improve adherence. Adherence to medication and lifestyle changes will be ensured through care managers and digital nudges, providing actionable, personalized insights and gamified competitions. Care managers will offer personalized insights and actionable recommendations to support patients in following their medication and lifestyle plans. Digital nudges, such as reminders and motivational messages, will be used to encourage patients to stay on track. Gamified competitions may also be introduced to motivate patients and enhance adherence to medication and lifestyle changes. The color-coded scoring system is designed to check adherence, and a personalized coach from Nerve Center will guide the patient on the right track based on the scores.

Emergency services: A cardiac emergency response system is added to this DTx solution to help manage any cardiac emergencies. Access to an early detection system (SOS button availability at all times for symptoms such as breathlessness and fatigue) that alerts doctors and family members and triggers emergency protocol is also added. Once activated, doctors and family members will be alerted, triggering the emergency protocol. Easy access to ambulances equipped to handle cardiac events and predetermined hospitals based on availability features will be provided through DTx.

Health mall: This unique feature is a one-stop shop that will access quality and affordable medicines prescribed by the doctor and auto-scheduled and guideline-recommended diagnostic tests at home. The prescribed medications and diagnostic tests will be directly delivered to the doorstep as recommended by the respective physician.

Caregiver involvement: To improve the involvement of caregivers in HF management, caregivers will be equipped with a dedicated application that receives alerts and monitors vital signs, keeping them informed about the patient's condition with real-time updates. In addition, there will be specially designed training modules for caregivers. These training modules will help enhance caregivers' abilities and knowledge in managing the patient's specific medical condition.

Potential Benefits of DTx in HF Management

Digital therapeutics in HF management has multiple potential benefits through which it will help doctors manage their HF patients better by reducing rehospitalizations and mortality. With the use of DTx, it is possible to attain a reduction in mortality rate by ~30% and readmission rates by 20% through comprehensive care management, including medication and vital adherence. DTx can provide improvement in the quality of life and exercise capability of patients through improved adherence, personalized coaching, and dedicated care managers. DTx provides an early warning system to avoid serious events, along with emergency services (e.g., ambulance). The use of DTx can make data on vitals and medication adherence available to cardiologists to help in medicine titrations and triaging of patient symptoms.

**Figure 1:**
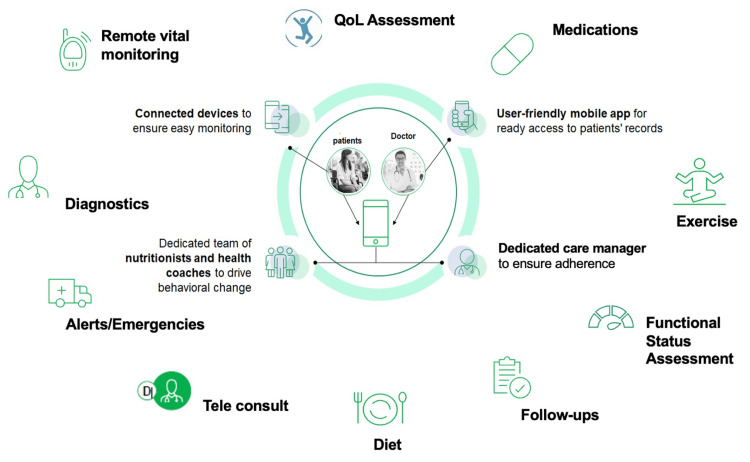
Potential of DTx for heart failure management in India DTx: digital therapeutics, QOL: quality of life

Figure 1 summarizes the important areas where DTx can contribute to the HF management program in India.

Patient-oriented benefits of DTx: Digital therapeutics in HF management can provide a comprehensive care package through specific diet plans, medication reminders, customized exercise plans, and vitals monitoring (including weight). In addition, DTx can provide patient education through comprehensive HF-related health education sessions and subsequently improve awareness about HF. The reduction in recurrent hospitalizations of DTx in HF brings numerous benefits to patients, including a significant reduction in the financial burden they face. All these patient-oriented benefits of DTx and providing support during emergencies ultimately can reduce the stress and anxiety levels among patients with HF.

Doctor-oriented benefits of DTx: Digital therapeutics in HF management can offer doctors a comprehensive platform that consolidates vital data, laboratory test results, patient history, and appointment schedules into a single system. This streamlined approach can simplify the assessment process and enhance efficiency for healthcare professionals. DTx can improve the ability of healthcare professionals to make informed decisions based on real-time data about symptoms and vitals obtained from the DTx platform. In brief, doctors can provide end-to-end patient care through DTx through a dedicated care team of managers and coaches. The utilization of DTx can yield significant benefits in efficient clinical practice. One such advantage is its potential to enhance GDMT compliance, improving patient outcomes. Additionally, DTx can also help reduce patient time spent in outpatient departments (OPDs), as it enables remote monitoring and provides timely interventions, minimizing the need for frequent in-person visits. This efficiency in clinical practice allows healthcare providers to optimize their resources and provide more personalized care to patients. The use of DTx by doctors can contribute to patient satisfaction, which in turn can promote increased patient retention and enhance patient lifetime value. Factors such as convenient and personalized care, remote monitoring, and timely interventions through DTx can significantly impact patient experience and overall satisfaction with their healthcare journey. As patients feel more engaged and supported in their treatment, they are more likely to continue their association with healthcare providers, improving patient retention rates. Moreover, the positive experiences and outcomes achieved through DTx can enhance the lifetime value of patients as they become more inclined to maintain long-term relationships with their healthcare providers. DTx has the potential to provide healthcare professionals with a future-ready practice.

Opinion of Experts About DTx for the Management of HF

The key insights suggested by experts include the following. Expert cardiologists suggested that the DTx solution should be user-friendly, easy to wear, and comfortable to use in everyday activities. These features will help healthcare professionals to promote its use and engage patients' participation. Experts opined that the best time to engage with patients regarding the use of DTx to reduce readmissions and improve QoL is during discharge and in OPD because two-thirds of readmissions are preventable with appropriate discharge planning [24]. Moreover, 30% of the patients get readmitted within the first week of hospital discharge and 60% within two weeks of discharge [24].

Add-ons in the DTx should include devices to measure saturated oxygen (SpO2), activity tracker, glucometer, electrocardiogram (ECG), diagnostic tests to determine electrolytes, renal function test, hemoglobin, blood urea nitrogen, iron profile, lipids, echo test, natriuretic peptide test (NT-proBNP), and teleconsultation (optional for doctors). Daily monitoring of blood pressure, resting heart rate, SpO2, blood sugar levels, and weight was suggested. However, ECG monitoring should be done every three months or when symptomatic. This is supported by global evidence such as research conducted by the German Federal Ministry of Education and Research [25] and Heart Failure Association of the European Society of Cardiology (ESC) [26].

The German Federal Ministry of Education and Research (TIM-HF2) used interventions such as algorithm-based guided care, devices for vitals (weighing scale, blood pressure device, ECG (two minutes or streaming), and SpO2), and a telemedical center to reduce unplanned cardiovascular hospital admissions by 26% and all-cause mortality by 30% [25].

The Heart Failure Association of the European Society of Cardiology used a similar intervention, i.e., digital therapeutics-based cardiac rehabilitation, to improve the exercise capacity and quality of life of HF patients [26].

Heart failure-specific symptoms such as shortness of breath, exercise intolerance or unusual pain, heart palpitations, feeling faint, swelling in your arms, legs, or abdomen, lack of appetite, fatigue, nausea, coughing and wheezing, and chest pain should be tracked with the help of reminders sent to the patient every week. Symptom assessment through a patient-facing mobile application, which is an integral part of the DTx program, could improve the monitoring of disease progression and QOL by changes in treatment or intervention with a targeted symptom. These opportunities can improve patient contact episodes to identify underlying problems and improve clinical management in a holistic fashion [27].

Among vitals, measuring SpO2, blood pressure, resting heart rate, blood sugar levels, and weight should be checked. Laboratory vitals such as hemoglobin, sodium, potassium, iron, thyroid, NT-proBNP, creatinine, urea, ECG, and echo should be monitored [28]. Handheld six-lead ECG should be considered daily for patients with AF or who are symptomatic. Salt and fluid intake monitoring is very important and should be done meticulously.

Alert management can be done through DTx. For all alerts, Nerve Center paramedics should reach out to the patient to advise them to visit the emergency room by assessing the severity of symptoms and off-range vitals if any (weight, BP, and HR).

Heart failure in India has emerged as an epidemic affecting 1% of the total population, with 10-12 million patients suffering from HF in India [2]. One million HF patients are diagnosed every year [2]. Hence, patient education and awareness become pivotal for better clinical outcomes. Patient education videos for essential topics (basics of HF as a disease, understanding your symptoms, limiting salt and fluids, tips to follow a low-sodium diet, monitoring your weight and body swelling, monitoring vitals apart from weight, diet plans, tips for following your diet, exercise, and activity, tips for exercising regularly, tips for taking all of your medicines as prescribed, managing other chronic conditions and their medications, managing feelings about heart failure, tips for family and caregiver, and living your best life with advanced HF) should be developed.

Relevant research studies and clinical trials

Telemonitoring allows patients to remotely provide digital health information to enhance and optimize their healthcare. This includes collecting data on symptoms, weight, heart rate, and blood pressure at regular intervals. The collected data is stored in an electronic health record and can guide patients, either directly or through healthcare professionals, in adjusting therapy or seeking further advice. Home telemonitoring (HTM) helps maintain the quality of care, enables quick access to care when necessary, reduces patient travel costs, and minimizes the need for frequent clinic visits [29]. The 2022 American Heart Association (AHA)/American College of Cardiology (ACC)/Heart Failure Society of America (HFSA) has identified studies on telehealth, digital health, applications, wearable technology, and artificial intelligence as evidence gaps and future research directions [28]. Table 1 states the 2021 ESC Guidelines for telemonitoring in the management of acute and chronic heart failure [30-32].

**Table 1:** 2021 ESC guidelines for telemonitoring in the management of acute and chronic heart failure CV, cardiovascular; ESC, European Society of Cardiology; HF, heart failure

Recommendation	Class	Level
Non-invasive home telemonitoring may be considered for patients with HF in order to reduce the risk of recurrent CV and HF hospitalizations and CV death [31].	IIb	B
Monitoring of pulmonary artery pressure using a wireless hemodynamic monitoring system may be considered in symptomatic patients with HF in order to improve clinical outcomes [32].	IIb	B

**Figure 2:**
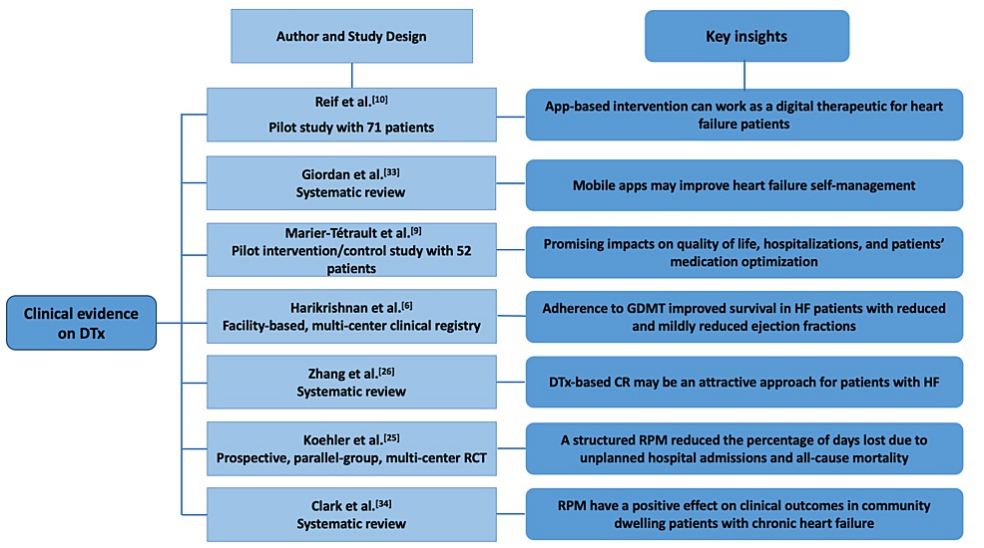
Recent clinical trials and evidence on benefits offered by DTx CR, cardiac rehabilitation; DTx, digital therapeutics; GDMT, guideline-directed medical therapy; HF, heart failure; RPM, remote patient monitoring

There is insufficient data to demonstrate the benefits of DTx in the management of HF. However, few studies in the past have successfully demonstrated the effectiveness of DTx in the management of HF (Figure 2) [6,9,10,25,26,33,34].

In the recent pilot study conducted on Indian patients with CAD (n=30), the efficacy of the DTx for HF was assessed over a three-month follow-up. Overall observations indicated that the use of DTx improved medication adherence and blood pressure control in patients with acute coronary syndrome (ACS) and/or post-percutaneous coronary intervention (PCI) [35].

Potential pitfalls and proactive strategies

It is essential to acknowledge and address several potential pitfalls and hazards to ensure the responsible integration of DTx technologies into healthcare systems. A primary concern revolves around the potential compromise of patient privacy [36]. To counteract this, the implementation of robust encryption technologies, strict adherence to data protection legislation, regular security audits, and comprehensive user education on data security practices are essential measures. Additionally, the reliability of monitoring systems poses a critical challenge, demanding rigorous validation methods, regular calibration, and the establishment of clear user guidelines. Interoperability issues with existing healthcare systems further underscore the importance of designing DTx solutions with interoperability in mind, adherence to industry standards, and collaborative efforts with healthcare IT providers. The effectiveness of DTx hinges on user adherence and engagement, necessitating a deliberate focus on user-centered design, the incorporation of behavioral scientific concepts, personalization, and the continuous refinement of engagement techniques. The scarcity of clinical evidence supporting DTx efficacy highlights the significance of thorough clinical studies, publication of outcomes, engagement with healthcare providers, and continuous updates based on emerging evidence. Addressing the digital divide, cybersecurity threats [36], and socioeconomic hurdles is equally vital to ensuring equal access and maintaining system integrity. By proactively handling these challenges, stakeholders can contribute to the responsible and effective integration of DTx into healthcare ecosystems.

## Conclusions

Digital therapeutics offers a promising avenue for improving HF management in India by enabling remote monitoring, enhancing patient engagement, and optimizing healthcare delivery. With careful consideration of challenges and collaborative initiatives, DTx has the potential to revolutionize HF care, leading to better outcomes and improved QOL for patients across the country. Remote consultations, virtual education, and guidance through digital platforms bridge the gap between patients and healthcare providers, ensuring continuous care and reducing the need for frequent hospital visits. Indeed, there is a need for collaborative efforts involving healthcare providers, policymakers, technology developers, and patient advocacy groups that are crucial for the successful implementation and integration of DTx into the healthcare system.

DTx for HF, India's sole evidence-based DTx solution, comprises a dedicated team of cardiologists, health coaches, care managers, and globally certified connected devices. This comprehensive platform offers support to patients with cardiac diseases, as well as those on their path to recovery following a cardiac event. DTx for HF has the potential to significantly enhance cardiac patients' vital signs and QOL.
